# Real-Life Data on the Safety of Pasireotide in Acromegaly: Insights from EudraVigilance

**DOI:** 10.3390/ph17121631

**Published:** 2024-12-04

**Authors:** Ioana Rada Popa Ilie, Carmen Maximiliana Dobrea, Anca Butuca, Calin Homorodean, Claudiu Morgovan, Andreea Loredana Vonica-Tincu, Felicia Gabriela Gligor, Steliana Ghibu, Adina Frum

**Affiliations:** 1 Department of Endocrinology, Faculty of Medicine, “Iuliu Haţieganu” University of Medicine and Pharmacy, 3-5 Louis Pasteur Street, 400349 Cluj-Napoca, Romania; ioana.ilie@umfcluj.ro; 2 Preclinical Department, Faculty of Medicine, “Lucian Blaga” University of Sibiu, 550169 Sibiu, Romania; anca.butuca@ulbsibiu.ro (A.B.); claudiu.morgovan@ulbsibiu.ro (C.M.); loredana.vonica@ulbsibiu.ro (A.L.V.-T.); felicia.gligor@ulbsibiu.ro (F.G.G.); adina.frum@ulbsibiu.ro (A.F.); 3 Medical Clinic No. 1, Internal Medicine Department, University of Medicine and Pharmacy “Iuliu Hatieganu”, 400006 Cluj-Napoca, Romania; 4 Interventional Cardiology Department, Cluj County Emergency Hospital, 400006 Cluj-Napoca, Romania; 5 Department of Pharmacology, Physiology and Pathophysiology, Faculty of Pharmacy, “Iuliu Haţieganu” University of Medicine and Pharmacy, 6A Louis Pasteur Street, 400349 Cluj-Napoca, Romania; steliana.ghibu@umfcluj.ro

**Keywords:** somatostatin receptor ligand, pasireotide, pharmacovigilance, EudraVigilance, real-world evidence

## Abstract

Background/Objectives: Pasireotide (PAS) is a somatostatin receptor ligand (SRL) used to treat acromegaly, a chronic condition caused by excess growth hormone. While it offers significant benefits as a second-line treatment for uncontrolled acromegaly, its use raises major concerns due to hyperglycemic side effects and gastrointestinal issues, the latter being similar to those seen with first-generation SRLs. The aim of this study is to evaluate the real-world evidence on adverse drug reactions (ADRs) reported for PAS in the EudraVigilance database, in comparison to other established drug-based therapies for acromegaly. Methods: A descriptive analysis and a disproportionality analysis were conducted. Results: The fewest individual case safety reports (ICSRs) and adverse drug reactions (ADRs) were reported for PAS, with 698 (4%) ICSRs and 1,647 (4%) ADRs, which is even lower than for pegvisomant (PEG), which had 1765 (11%) ICSRs and 4842 (10%) ADRs. Both PAS and lanreotide (LAN) exhibited the lowest proportion of cases classified as serious. Among the total reported ADRs, those categorized as “Metabolic and nutrition disorders” were most frequent and severe for PAS (PAS—17.5% vs. OCT—4.6%, LAN—4.5%, and PEG—2.7%). Additionally, PAS demonstrated a higher likelihood of reporting endocrine disorders, which were frequently classified as serious, as well as stones affecting the hepatobiliary system compared to other drugs. Conclusions: Although PAS had the fewest ICSRs and ADRs, and less frequent serious ADRs, it had more reports frequently classified as serious in the “Metabolism and Nutrition Disorders” category (including events such as elevated blood glucose levels or diabetes) and “Endocrine Disorders” category compared to other SRLs and PEG. Furthermore, there was a higher likelihood of reporting hepatobiliary stones with PAS compared to OCT and PEG. This highlights the importance of adequately monitoring glycemic control and the biliary tract through ultrasound at the initiation and during follow-up of PAS therapy. Improved monitoring and reporting of these ADRs could enhance care for patients with acromegaly.

## 1. Introduction

Pasireotide (PAS) is a novel second-generation (sg) somatostatin receptor ligand (SRL) approved for the treatment of acromegaly and Cushing’s disease (CD), offering a new therapeutic option for patients. Acromegaly is a rare disorder caused by a growth hormone (GH) secreting pituitary adenoma, also known as a somatotropinoma, in over 99% of cases. This results in excess GH and, consequently, the overproduction of insulin-like growth factor-I (IGF-I), which drives the majority of the condition’s manifestations. Acromegaly is typically a slow-progressing, chronic, systemic disorder associated with many cardiovascular, metabolic, and neoplastic complications as well as increased mortality if not adequately treated. Although tumor resection via transsphenoidal surgery is the optimal primary treatment in most patients and in most centers, remission rates vary depending on the size of the adenoma, the invasiveness of the tumor, and the expertise of the center [1,2]. Because biochemical remission remains the strongest predictor of patient outcomes [3], patients who do not achieve biochemical control after surgery should be managed with medical therapy aiming to obtain and maintain serum IGF-I levels in the mid to upper half of the age-related reference range [4].

The development of somatostatin receptor ligands (SRLs), also known as somatostatin analogs (SSAs), was an important milestone in the medical treatment of acromegaly. Native somatostatin (SST) and SSAs elicit their biological effects by activating somatostatin receptors (SSTRs). There are five distinct SSTRs, types 1–5 [5]. Somatotropinomas predominantly express SSTR types 2 and 5, each expressed in approximately 90% of GH-secreting pituitary adenomas. They have been useful for tumor-targeted pharmacotherapy with the first-generation (fg) SRLs octreotide (OCT) and lanreotide (LAN) [5,6,7,8,9,10,11]. However, overall, OCT and LAN are effective in approximately 20–70% of patients. Consequently, a subset of patients (nonresponders) require other treatment options for more efficacious disease control. Hence, the development of new medical therapies remains essential [12]. On the other hand, corticotroph adenomas and corticotropin (also adrenocorticotropin) (ACTH)-secreting pituitary adenomas also express SSTRs, predominantly the SSTR5 subtype. Activation of this subtype inhibits ACTH secretion, which formed the basis for investigating it as a potential therapeutic target for CD [13,14,15,16].

The novel SRL PAS binds with a high affinity to all SSTR subtypes, with the exception of SSTR4, and with higher affinity to SSTR type 5 than type 2, unlike fg-SRLs [11,17,18]. Because of its broader binding profile, PAS has been suggested to have greater clinical efficacy in acromegaly than fg-SRLs and to be efficacious in CD [19,20]. Pasireotide (brand name Signifor), typically administered as a subcutaneous injection twice daily, was first approved by the US Food and Drug Administration (FDA) and by the European Medicines Agency (EMA) in 2012 for the treatment of Cushing’s disease in patients who cannot undergo surgery or for whom surgery has not been successful. In 2014, the approval was expanded to include the PAS long-acting release (LAR) formulation (Signifor LAR), a monthly intramuscular injection, for the treatment of acromegaly and, more recently, Cushing’s disease. Octreotide LAR and LAN autogel (LAN-ATG) are still recommended as first-line medical therapies in the guidelines, due to their favorable risk/benefit profiles [21], whereas PAS is usually used in acromegaly patients resistant to fg-SRLs. OCT, necessitating multiple (2–3) subcutaneous injections, was the first one developed [22]. However, long-acting SRL formulations (OCT-LAR administered via intramuscular injection and a pre-filled syringe with LAN-ATG for deep subcutaneous injection), both typically given every 4 weeks, are much more useful in clinical practice [11].

The GH receptor antagonist pegvisomant (PEG), approved by the FDA in 2003 and the EMA in 2004 for the treatment of acromegaly, can normalize IGF-I concentrations in roughly 60–97% of patients [23,24]; however, it does not reduce GH concentrations or tumor volume. Administered as a monotherapy or in combination with SRLs, through daily subcutaneous injections, it is generally used as second-line therapy in patients who do not achieve biochemical control with maximal doses of SRL, as well as in those with no clinically relevant residual tumor [21].

Given the overlapping indications and patient populations, a comparative analysis of the adverse drug reactions (ADRs) associated with PAS and other established drugs in the management of acromegaly is crucial. Moreover, the chronic nature of acromegaly necessitates long-term use of these therapies, requiring vigilant monitoring to ensure that patients achieve sustained biochemical control without experiencing significant side effects. SRLs, while effective, may be associated with gastrointestinal disturbances, biliary tract abnormalities, or hyperglycemia and impaired glucose tolerance (IGT) [5], whereas PEG can lead to liver enzyme elevations and injection site reactions [25]. Regular monitoring helps mitigate these risks, ensuring that treatment remains both effective and safe, ultimately improving patients’ outcomes and quality of life. The monitoring and reporting of ADRs are often consolidated in pharmacovigilance databases like EudraVigilance (EV), which plays a vital role in ensuring drug safety. EV is the European Union’s (EU) centralized system for managing and analyzing information on suspected ADRs, providing access to real-time data. This system ensures that drugs used across the EU, including those for chronic diseases like acromegaly, remain safe and effective, ultimately improving patient outcomes by minimizing the risks of ADRs. EV collects reports from a wide range of sources, including healthcare professionals, patients, and pharmaceutical companies, making it one of the most extensive databases for ADRs [26].

A few studies have examined the efficacy and safety of PAS in real-world settings. While these studies have confirmed the efficacy of PAS-LAR, with a good biochemical response in 20–54% of acromegaly patients, they also reveal significant worsening of glucose metabolism, which may require early and aggressive intervention—contrary to findings from previous clinical trials [27,28,29,30,31]. Real-world studies are crucial for tailoring acromegaly treatment and identifying patients who are most likely to benefit from PAS-LAR treatment [32,33].

This article aims to analyze the pharmacovigilance data of PAS based on real-life data from EV and to compare its safety profile with those of OCT, LAN, and PEG, contributing to a better understanding of the safety considerations for these treatments in patients with acromegaly.

## 2. Results

### 2.1. Descriptive Analysis

**Figure 1:**
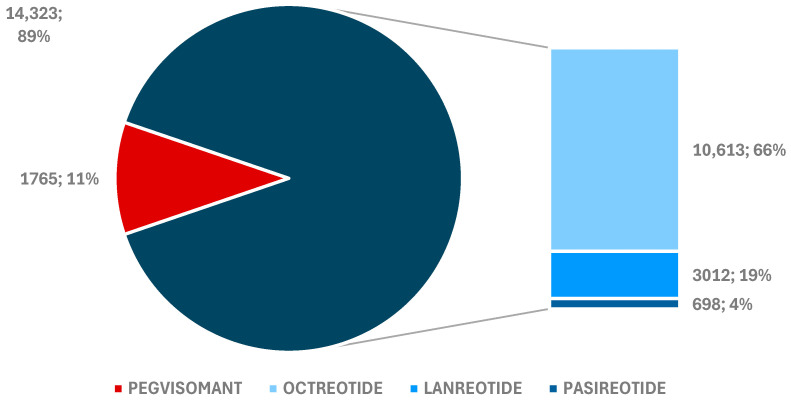
Total individual case safety reports (ICSRs) reported for drugs used in acromegaly treatment.

According to Figure 1, 14,323 reports (89%) for somatostatin receptor ligands (SRLs) were submitted to the EV database, compared to only 1765 ICSRs (11%) for PEG. The majority of reports were registered for OCT (*n* = 10,613), while the fewest were for PAS (*n* = 698).

**Figure 2:**
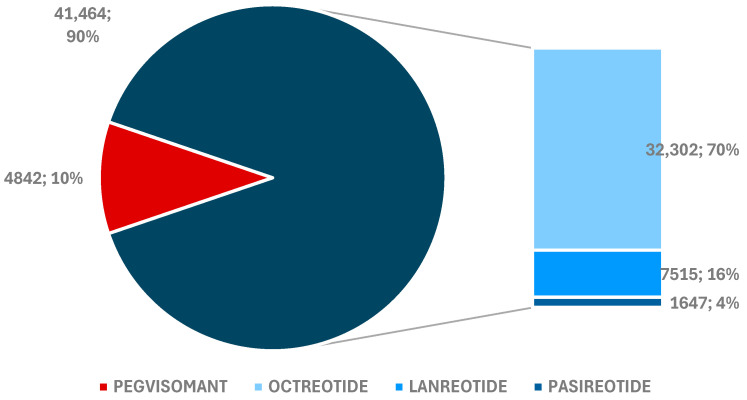
Total ADRs reported for drugs used in acromegaly treatment.

There are similarities between the proportions of adverse drug reactions (ADRs) and individual case safety reports (ICSRs). Thus, the highest number of ADRs were reported for OCT (*n* = 32,302) and the lowest for PAS (*n* = 1647) (Figure 2).

**Figure 3:**
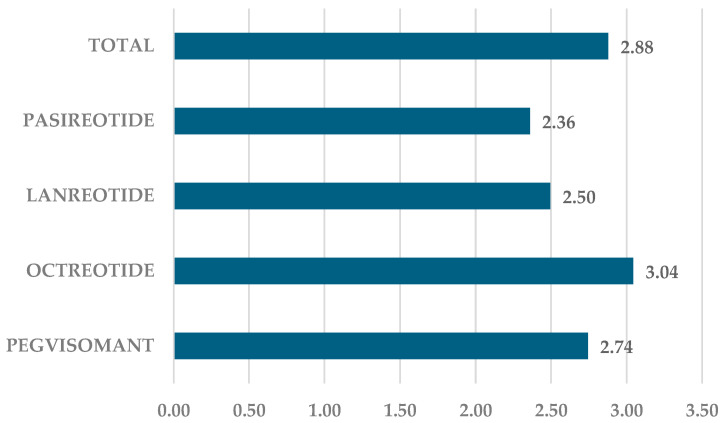
The proportions of ADRs reported per ICSR.

For all four drugs, an average of 2.88 ADRs were reported per ICSR. The highest number of ADRs per ICSR was for OCT (*n* = 3.04), and the lowest was for PAS (*n* = 2.36) (Figure 3). Additionally, for LAN (*n* = 2.50) and PEG (*n* = 2.74), the number of ADRs per ICSR was below the group average.

An analysis of the demographic characteristics of the patients reveals some similarities between PAS and PEG in the distribution of reports by patient age (Table 1). Unlike OCT (33.68%) and LAN (38.84%), similar frequencies of ICSRs were recorded for both PAS (55.44%) and PEG (58.02%) in the 18–64 age group. In the 65–85 age category, PAS had a lower frequency (14.76%) compared to the other drugs (OCT: 22.12%; LAN: 26.10%; and PEG: 24.48%). Furthermore, ADRs were reported more frequently in females for PAS (62.32%) compared to the other drugs (OCT: 49.65%; LAN: 52.22%; and PEG: 57.34%). Conversely, the lowest frequency of ICSRs in males was recorded for PAS (28.94%). Most reports for LAN (59.79%) and PAS (56.30%) originated from European Economic Area (EEA) countries. Healthcare professionals (HPs) were the primary reporters of signals in the EV database for all drugs, particularly for OCT (86.08%) and PAS (82.81%).

**Figure 4:**
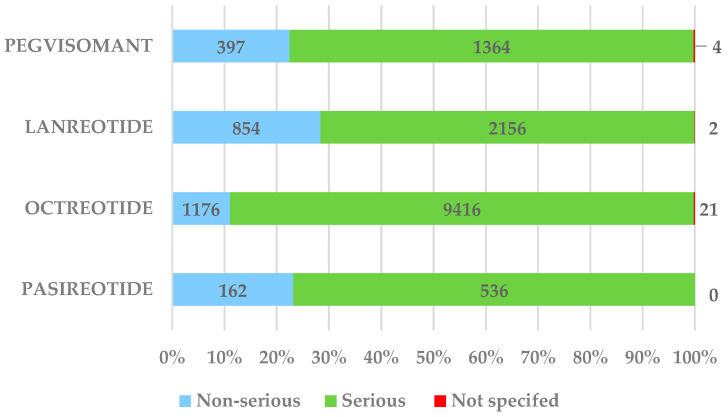
Distribution of cases by seriousness.

Approximately 76.8% of cases reported for PAS were classified as serious, a lower frequency compared to OCT (88.72%) and similar to PEG (77.28%) (Figure 4).

Regarding the distribution of cases by System Organ Class (SOC), several differences were notable (Supplementary Materials—Table S1):

- Compared to all other drugs, a lower frequency of cases reported for PAS was observed in the following SOCs: “Cardiac disorders” (1.9%), “Vascular disorders” (1.8%), “General disorders and administration site conditions” (14.3%), “Infections and infestations” (2.6%).
- A higher frequency of signals was reported for patients treated with PAS in various SOCs: “Endocrine disorders” (PAS—3.2% vs. OCT—1.3%, LAN—1.4%, and PEG—1.7%), “Metabolism and nutrition disorders” (PAS—17.5% vs. OCT—4.6%, LAN—4.5%, and PEG—2.7%), “Renal and urinary disorders” (PAS—2.5% vs. OCT—1.9%, LAN—1.6%, and PEG—1.5%), “Psychiatric disorders” (PAS—1.3% vs. OCT—2.9%, LAN—2.2%, and PEG—2.9%), etc.
- Similar frequencies were reported for all drugs in the “Hepatobiliary disorders” SOC.
- Compared to PEG, a higher frequency of signals in the “Gastrointestinal disorders” SOC was registered for PAS (10.0% vs. 6.7%).

**Figure 5:**
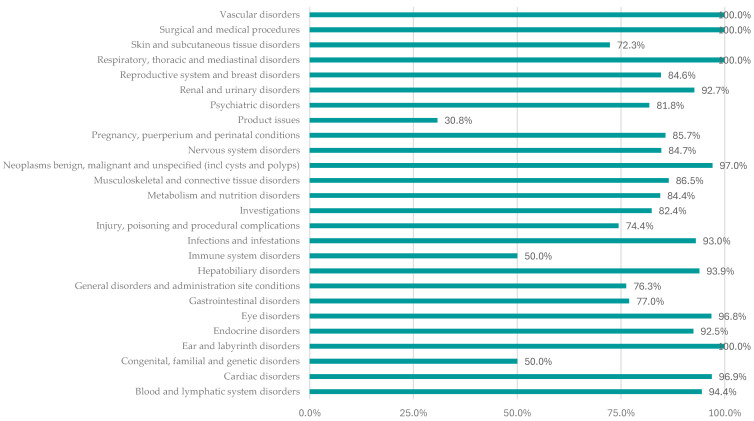
Distribution of serious ADRs by SOC for pasireotide.

According to Figure 5, a high frequency of serious ADRs was reported for PAS in the following SOCs: “Vascular disorders” (100%), “Cardiac disorders” (96.9%), “Hepatobiliary disorders” (93.9%), “Endocrine disorders” (92.5%), and “Metabolism and nutrition disorders” (84.4%). On the other hand, the frequency of serious ADRs was lower for gastrointestinal disorders (77.0%).

### 2.2. Disproportionality Analysis

**Figure 6:**
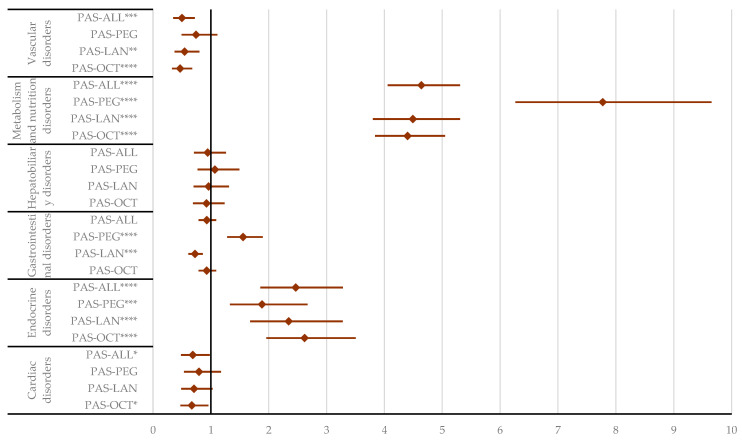
Disproportionality analysis in selected SOCs. ALL—all drugs except pasireotide; LAN—lanreotide; OCT—octreotide; PAS—pasireotide; PEG—pegvisomant. * *p* < 0.05; ** *p* ≤ 0.01; *** *p* ≤ 0.001; **** *p* ≤ 0.0001.

In the “Cardiac disorders” SOC, PAS had a lower reporting probability compared to all other acromegaly treatments (reporting odds ratio (ROR): 0.69; 95% CI: 0.48–0.98) and OCT specifically (ROR: 0.67; 95% CI: 0.47–0.96). Similarly, in the “Vascular disorders” SOC, PAS had a lower probability of reporting than OCT (ROR: 0.47; 95% CI: 0.33–0.68), LAN (ROR: 0.55; 95% CI: 0.37–0.80), and other acromegaly treatments (ROR: 0.50; 95% CI: 0.35–0.72). For “Gastrointestinal disorders”, PAS was reported less frequently than LAN (ROR: 0.72; 95% CI: 0.61–0.86) but more often than PEG (ROR: 1.56; 95% CI: 1.28–1.90). In contrast, PAS had a higher probability of reporting endocrine, metabolism, and nutrition disorders compared to other drugs. No statistical differences were found between PAS and other comparators for ADRs in the “Hepatobiliary disorders” SOC (Figure 6, Supplementary Materials—Table S2).

**Figure 7:**
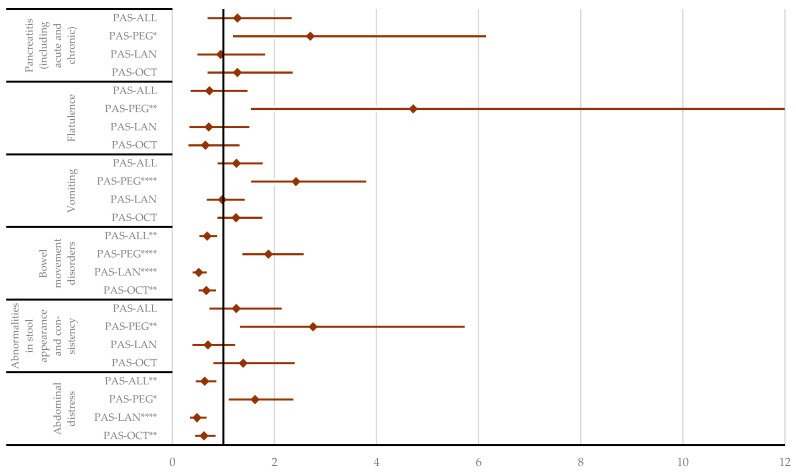
Disproportionality analysis for gastrointestinal ADRs. ALL—all drugs except pasireotide; LAN—lanreotide; OCT—octreotide; PAS—pasireotide; PEG—pegvisomant. * *p* < 0.05; ** *p* ≤ 0.01; **** *p* ≤ 0.0001. According to the results in Figure 8, a higher probability of reporting stones affecting the hepatobiliary system was observed for PAS compared to OCT (ROR: 1.50; 95% CI: 1.04–2.17), PEG (ROR: 2.11; 95% CI: 1.32–3.35), and all other drugs (ROR: 1.51; 95% CI: 1.05–2.17). Other HLTs from the hepatobiliary disorders group could not be evaluated due to the low number of ICSRs reported for PAS (Supplementary Materials—Table S4).

Compared to PEG, PAS has a higher probability of reporting gastrointestinal ADRs (Supplementary Materials—Table S3) from various High-Level Terms (HLTs), including (i) abdominal distress (ROR: 1.62; 95% CI: 1.11–2.37), (ii) abnormalities in stool appearance and consistency (ROR: 2.76; 95% CI: 1.33–5.73), (iii) bowel movement disorders (ROR: 1.88; 95% CI: 1.38–2.58), (iv) flatulence (ROR: 4.72; 95% CI: 1.54–14.45), (v) vomiting (ROR: 2.42; 95% CI: 1.55–3.80), and (vi) pancreatitis (ROR: 2.71; 95% CI: 1.19–6.15). Conversely, PAS has a lower probability of reporting abdominal distress than LAN (ROR: 0.49; 95% CI: 0.35–0.67) and OCT (ROR: 0.62; 95% CI: 0.45–0.85). Bowel movement disorders were also reported to have a lower probability for PAS compared to OCT (ROR: 0.67; 95% CI: 0.52–0.86) and LAN (ROR: 0.52; 95% CI: 0.40–0.67) (Figure 7).

**Figure 9:**
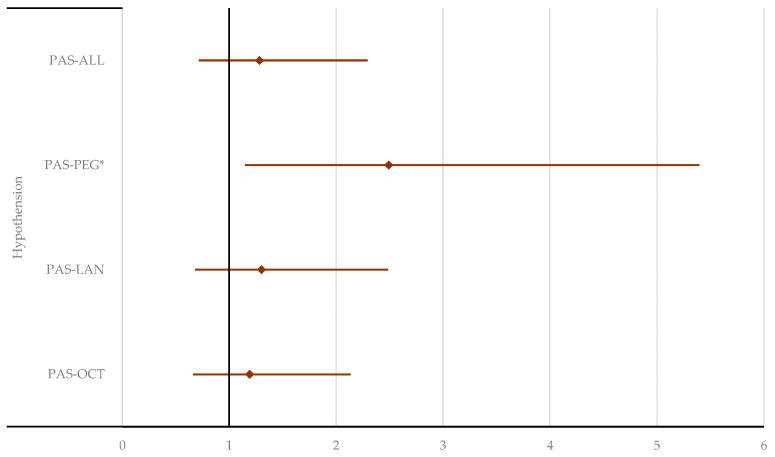
Disproportionality analysis for hypotension. ALL—all drugs except pasireotide; LAN—lanreotide; OCT—octreotide; PAS—pasireotide; PEG—pegvisomant. * *p* < 0.05.

In the “Cardiac disorders” SOC, the low number of ICSRs reported for PAS (*n* < 5) does not allow for the evaluation of reporting probability, except for arrhythmia. However, no significant differences were observed for this HLT. A similar situation occurred for “Vascular disorders” (Supplementary Materials—Table S5), where the ROR could only be calculated for hypotension. As shown in Figure 9, hypotension was more likely to be reported with PAS than PEG (ROR: 2.49; 95% CI: 1.15–5.40).

**Figure 10:**
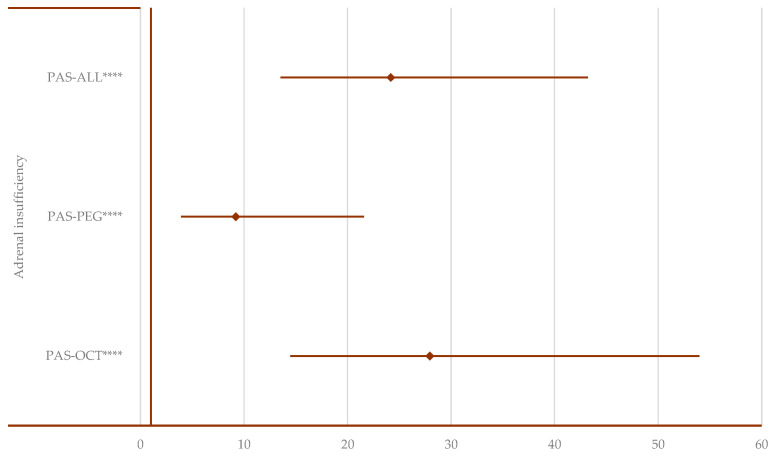
Disproportionality analysis for adrenal insufficiency. ALL—all drugs except pasireotide; OCT—octreotide; PAS—pasireotide; PEG—pegvisomant. **** *p* ≤ 0.0001.

In the “Endocrine disorders” SOC, a disproportionate signal was observed only for adrenal insufficiency (Supplementary Materials—Table S6). Adrenal insufficiency was reported more frequently for PAS compared to OCT (ROR: 27.96; 95% CI: 14.48–54.00), PEG (ROR: 9.20; 95% CI: 3.92–21.58), and all other drugs used for acromegaly (ROR: 24.18; 95% CI: 13.53–43.21) (Figure 10).

**Figure 11:**
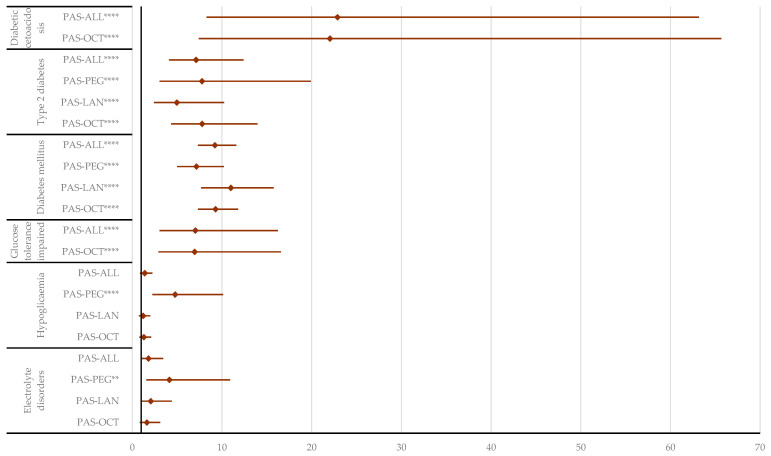
Disproportionality analysis of metabolic and nutritional disorders. ALL—all drugs except pasireotide; LAN—lanreotide; OCT—octreotide; PAS—pasireotide; PEG—pegvisomant ** *p* ≤ 0.01; **** *p* ≤ 0.0001.

In the “Metabolism and nutrition disorders” SOC, no relevant signals (*n* < 5) were registered for following HLTs hypoalbuminemia, hypercholesterolemia, gout, and type 1 diabetes, preventing an evaluation of disproportionality for these. However, disproportionate signals were observed for PAS regarding electrolytic disorders, diabetic ketoacidosis, glucose tolerance impairment, diabetes mellitus, and type 2 diabetes (Supplementary Materials—Table S7). Electrolytic disorders (ROR: 4.15; 95% CI: 1.58–10.93) and hypoglycemia (ROR: 4.78; 95% CI: 2.25–10.13) were reported to be more likely for PAS compared to PEG, and diabetic ketoacidosis was reported more frequently compared to OCT (ROR: 22.04; 95% CI: 7.40–65.67) and all other drugs (ROR: 22.88; 95% CI: 8.29–63.17). Diabetes mellitus and type 2 diabetes were also reported more frequently for PAS than for the other comparators. Additionally, PAS had a higher probability of reporting IGT compared to OCT (ROR: 6.96; 95% CI: 2.92–16.58) and all other drugs used in the treatment of acromegaly (ROR: 7.04; 95% CI: 3.05–16.24) (Figure 11).

## 3. Discussion

In this study, we assessed the safety profile of PAS based on real-world data from the EV platform and compared its ADRs with those of the other drugs used in the medical management of acromegaly: OCT, LAN, and PEG. Although cabergoline is sometimes used off-label in patients with acromegaly, it was not included in our comparative analysis. Cabergoline, a dopamine agonist, is not approved for use in acromegaly, but it is occasionally prescribed for patients with mildly elevated IGF-I levels after surgery or as an add-on therapy when biochemical control is not achieved with the maximum doses of SRLs or PEG [21]. PAS-LAR is an sg-SRL approved as a second-line medical therapy in patients with acromegaly, typically initiated after the failure of fg-SRLs [34,35]. The most common side-effects of PAS treatment in clinical studies on acromegaly include hyperglycemia, diarrhea, diabetes, and cholelithiasis [36,37].

The findings from our study showed that the fewest ICSRs and ADRs reported in EudraVigilance were for PAS, significantly fewer than those reported for LAN, PEG, and especially OCT. A similar trend was observed for the number of ADRs per ICSR. Additionally, the frequency of reported serious ADRs was lower for PAS and LAN compared to PEG and especially OCT. PAS is the most recent SRL introduced to the market, while OCT is the oldest. However, the spectrum of side effects of PAS is similar to those of fg-SRLs [11].

There are very few studies that directly compare the safety of PAS-LAR with other SRLs [20,38]. Moreover, in most studies, patients with inadequately controlled acromegaly are switched from OCT or LAN to PAS. Accordingly, reports from EV are largely consistent with the literature, showing that ADR frequencies are higher in patients naive to SRL treatment (40–45%) who were initiated on SRLs compared to those previously treated with SRLs (5–20%) [12,20,37].

We found one study that made a head-to-head comparison of PAS-LAR and OCT-LAR in drug-naive patients with acromegaly. The results revealed lower rates of ADRs, excluding hyperglycemia and diabetes mellitus, in the PAS-LAR group compared to the OCT-LAR group [20]. Additionally, lower rates of serious adverse events (Common Terminology Criteria (CTC) grade 3–4), excluding hyperglycemia and diabetes mellitus, were observed in the PAS-LAR group compared to the OCT-LAR group [20]. Similar findings were reported for the extension phase of a large Phase III study [38]. PAS provided clinical benefits and was well tolerated for more than 11 years of treatment in acromegaly patients, most of whom were resistant to fg-SRLs, as revealed by a single-center retrospective analysis of efficacy and safety data. Overall, PAS was generally associated with mild and manageable ADRs [34]. Moreover, safety data from an expanded treatment protocol assessing PAS-LAR in a real-world clinical setting showed a low incidence of grade ≥3 or serious treatment-emergent adverse events, occurring in 25.0% of patients, with 11 cases suspected to be related to PAS-LAR. Only 11.4% of patients discontinued treatment due to ADRs, while 45.5% experienced hyperglycemia-related ADRs [10]. The latest meta-analysis, encompassing 12 studies (11 with non-duplicated samples) involving a total of 409 patients, assessed the real-world effectiveness and safety of PAS-LAR for acromegaly. The findings indicated higher IGF-I control rates than previously estimated from randomized controlled trials. However, this benefit was accompanied by significant increases in glucose levels, HbA1c, and the prevalence of type 2 diabetes mellitus, with a standardized mean difference of 11.5% (95% CI: −17.5 to −5.5) [39]. Another very recent systematic review and meta-analysis of nine studies involving 590 participants identified gastrointestinal disturbances as the most common adverse event, affecting 31.26% of acromegaly patients treated with PAS. Hyperglycemia was the second most frequently reported issue, impacting 29.55% (95% CI: 21.80–37.29) of patients. Notably, the incidence of new-onset diabetes mellitus significantly increased, with a rate of 23.36% (95% CI: 19.58–27.13) [40].

In general, ADRs occur in 46–94% of patients treated with OCT-LAR, with almost all being treatment-related [41]. Grade 3 or 4 toxicity is rare, according to the majority of studies (or severe ADRs are not treatment-related) [42]. An open, randomized multicenter study compared the efficacy and safety of OCT-LAR and LAN (LAN-SR, Somatuline^®^, Ipsen Biotech) in 125 acromegalic patients previously treated with LAN-SR. Although the study design did not allow for a direct comparison of systemic tolerability, 70 patients (56%) on OCT-LAR and 12 patients (44%) on LAN-SR reported at least one adverse event. The investigators suspected treatment-related ADRs in 24 patients (19%) receiving OCT-LAR and two patients (7%) receiving LAN-SR [43].

According to the present study, similar frequencies of ICSRs were recorded for both PAS (55.44%) and PEG (58.02%) in the 18–64 age group. For PAS, the frequency was higher compared to the 65–85 age group (14.76%). This difference is probably explained by the more frequent use of PAS and PEG in younger acromegaly patients. Both PAS and PEG are generally recommended in acromegaly patients whose condition is inadequately controlled by OCT-LAR or LAN, and some of the main clinical predictors of fg-SRL resistance recognized in the literature include male sex and young age [44,45,46].

ADRs have been reported more frequently in females for PAS. In most studies, there is an equal distribution of acromegaly prevalence between males and females, while Cushing’s disease (CD) has significantly higher preponderance among females with incidence rates that approach 3–8 times those in males [47]. This could explain the use of this preparation more in women and thus the difference in the reporting of ADRs in favor of the female sex. Women are nearly twice as likely as men to experience ADRs across all drug classes and are more frequently hospitalized due to ADRs [48,49]. Additionally, impaired glucose metabolism and diabetes mellitus, well-known complications of acromegaly, are reported to be more prevalent in females [50,51]. Furthermore, female gender is one of the most important risk factors for gallstone formation [52], a common complication in PAS treatment, alongside hyperglycemia-related events [36,37].

Compared to other drugs, a lower frequency of cases was reported for PAS in the following SOCs: “Cardiac disorders” and “Vascular disorders”. In fact, among the total cases reported, those in these two SOCs are the least common for PAS, but nearly all are classified as severe. Cardiovascular adverse effects are rarely reported in studies involving SSRLs. SSTRs 1, 2, 4, and 5 are expressed in the human heart [53]. The following cardiovascular ADRs and their incidences are derived from the product labeling of PAS: hypertension (8% to 15%), peripheral edema (10% to 14%), atrioventricular block (long-acting release: 6%), hypotension (6% to 8%), a prolonged QT interval on ECG (1% to 6%), and sinus bradycardia (3% to 10%) [54]. One patient with Cushing’s disease and a history of hypertension and uncontrolled hypothyroidism experienced a serious adverse event related to QT interval prolongation (Bazett’s formula) while on PAS treatment [13]. Additionally, there was one case of fatal myocardial infarction in a patient from the OCT-LAR group in a trial comparing PAS-LAR with OCT-LAR in treatment-naive patients with acromegaly [20]. Patients with acromegaly frequently present with a prolonged QT interval [55,56,57], and a recent study on CD found QT interval prolongation in males [58]. Since endogenous SST has been found to prolong QT intervals [59], the use of SRLs in treating CD or acromegaly may further increase the risk of cardiac arrhythmias in these patients. Bradycardia-related adverse events in Phase III clinical trials were reported in 3% to 8% of patients treated with PAS-LAR, compared to 0% in active controls (patients on fg-SRLs) [20,37]. We identified one case of venous thromboembolism (a serious adverse event) with PAS-LAR [34] and one case of fatal pulmonary embolism with OCT-LAR in acromegaly patients [43]. In CD, which is associated with a hypercoagulable state, two patients developed pulmonary embolism during the first month of PAS monotherapy [60].

The disproportionality analysis showed a higher probability of reporting hypotension with PAS compared to PEG, but no significant difference when compared to OCT or LAN. Additionally, no statistically significant difference was found for cardiac arrhythmias. According to the product labeling, hypertension, but not hypotension, is listed as an adverse reaction observed in clinical trials with PEG [61]. Long-term PEG therapy not only normalized IGF-I in a large proportion of patients with acromegaly, but also improved cardiac and respiratory comorbidity. However, mean systolic and diastolic blood pressure did not change significantly in patients who were normotensive or in those who were hypertensive at baseline [62].

As expected, based on its mechanism of action, a higher frequency of signals was reported for patients treated with PAS in the “Endocrine disorders” SOC, and particularly in the “Metabolism and nutrition disorders” SOC, compared to subjects treated with OCT, LAN, and PEG. These events are frequently classified as serious. Moreover, the disproportionality analysis showed that PAS had a higher probability of reporting endocrine, metabolism, nutrition, and endocrine disorders than the other drugs. Acromegaly is clinically linked to metabolic complications, with up to 50% of patients having prediabetes or diabetes [63,64]. Consequently, understanding the effects of PAS-LAR on glucose metabolism is crucial, both in clinical trials and in routine clinical practice.

A report from the first long-term extension phase of a large Phase III study revealed that 112 patients (62.9%) in the PAS-LAR arm experienced a hyperglycemia-related adverse event (including terms such as hyperglycemia, elevated glycated hemoglobin, diabetes mellitus, etc.), compared to 45 patients (25.0%) in the OCT LAR arm [38]. The results from a single-center study confirmed a significant worsening of glucose metabolism during treatment with PAS-LAR in acromegaly patients resistant to fg-SRLs. After 12 months, 11 patients (42.3%) were diabetic, 15 patients (57.7%) were prediabetic, and 1 patient (3.8%) had normal metabolic status compared to baseline, where 4 patients (15.4%) were diabetic, 19 (73.1%) were prediabetic, and 3 (11.5%) had normal metabolic status [29]. Other Phase III studies evaluating the efficacy and safety of PAS-LAR in acromegaly patients have also reported higher rates of hyperglycemia-related adverse events, including severe cases, in those treated with PAS-LAR compared to OCT-LAR or LAN-ATG [20,37]. Across the two studies, a total of thirteen patients (eight (4.5%) and five, respectively) discontinued PAS-LAR treatment due to a serious ADR, with PAS-induced hyperglycemia or diabetes being the most common reason [20,37].

The hyperglycemic effect of PAS can be explained by the drug’s binding-affinity profile. SSTRs are also expressed in pancreatic islet cells. Glucagon-producing alpha cells predominantly express SSTR2, whereas insulin-producing beta cells mainly express SSTR2 and SSTR5 [11,12]. Previous studies in healthy individuals demonstrated that PAS inhibits the secretion of insulin, glucagon-like peptide 1 (GLP-1), and glucose-dependent insulinotropic peptide (GIP), whereas the drug’s inhibitory effect on glucagon secretion is only modest, all of which may contribute to carbohydrate metabolism disturbances, a rise in blood glucose levels, and the development of IGT or diabetes [65].

In the pituitary gland, SST inhibits the secretion of several hormones: GH, prolactin, thyroid-stimulating hormone (TSH), and ACTH [66]. LAN treatment may potentially cause hypothyroidism [67]. Due to its broader receptor binding profile, PAS-LAR is expected to result in a higher incidence of ADRs such as hypothyroidism, adrenal insufficiency, and other metabolic disturbances compared to fg-SRLs. However, clinical trials have shown that both PAS-LAR and fg-SRLs have rarely been associated with adrenal insufficiency or hypothyroidism [5,20,37]. That said, cases of hypocortisolism-related ADRs (e.g., adrenal insufficiency, decreased blood cortisol, etc.) have been reported in patients with CD effectively treated with PAS [68]. The disproportionality analysis revealed that adrenal insufficiency was reported more frequently for PAS compared to OCT, PEG, and all other drugs used for acromegaly. However, given the potential for hormonal suppression, it is important to monitor all pituitary hormones in patients receiving PAS-LAR therapy.

Hypoglycemia is another important adverse event associated with PAS use in acromegaly, with reported frequencies ranging between 3% and 13.6% [11]. As mentioned, PAS modulates the secretion of glucagon, insulin, and incretin hormones, and lowers GH levels, which may lead to an overall decrease in blood glucose. Hypoglycemia has also been reported in rare isolated cases with PEG, particularly in patients with pre-existing conditions like diabetes. However, increased glycated hemoglobin and diabetes were observed in 12 subjects on PEG therapy in the ACROSTUDY [24]. In the “Metabolism and Nutrition Disorders” SOC, the following two HLTs—electrolytic disorders and hypoglycemia—were reported to be more likely with PAS compared to PEG. Electrolytic disorders (e.g., hypokalemia, hyponatremia, hypocalcemia, hypercalcemia) were reported more frequently with PAS compared to PEG, likely in relation to vomiting, diarrhea, diabetic pancreatitis, and pancreatolithiasis, which are commonly associated with PAS use. We identified one report of severe hyponatremia in a Phase II trial evaluating the efficacy and safety of PAS in acromegaly [69]. In the gastrointestinal system, SST suppresses the secretion of several hormones, including cholecystokinin, gastric inhibitory peptide, gastrin, motilin, neurotensin, secretin, glucagon, insulin, and pancreatic polypeptide. Additionally, it inhibits the exocrine activity of the gastrointestinal mucosa, salivary glands, and liver; modulates gastrointestinal absorption and motility; and reduces portal pressure. SST is also known to inhibit hepatic bile secretion and gallbladder emptying [11].

The frequency of signals in the “Gastrointestinal Disorders” SOC was similar for the three SRLs PAS, OCT, and LAN. In this category, the percentage of serious ADRs was lower than in other categories of interest. As expected, based on their mechanism of action, PAS was reported more frequently than PEG for “Gastrointestinal Disorders” (10.0% vs. 6.7%). The disproportionality analysis also revealed higher reporting rates (PAS vs. PEG) for various HLTs, including abdominal distress, abnormalities in stool appearance and consistency, bowel movement disorders, flatulence, vomiting, and pancreatitis. However, the analysis found that PAS was reported less frequently compared to fg-SRLs for “Gastrointestinal Disorders” abdominal distress, and bowel movement disorders.

Mild gastrointestinal disturbances were reported in the Phase II randomized, open-label, crossover, multicenter PAS (SOM230) study [69]. Adverse events suspected to be related to the study drug during PAS treatment included nausea (25%), diarrhea (22%), abdominal pain (12%), and flatulence (10%) [69]. An extension of the study revealed even higher rates of these ADRs (diarrhea (46.7%), nausea (33.3%), abdominal pain (20%), and flatulence (20%)) [70]. A direct comparison of PAS-LAR and OCT-LAR in drug-naive acromegaly patients showed that the PAS-LAR group experienced lower rates of gastrointestinal ADRs compared to the OCT-LAR group [20]. Similar results were reported in the extension phase of a large Phase III study, with diarrhea (45% vs. 39.9%), abdominal pain (24.4% vs. 18.5%), nausea (22.8% vs. 15.2%), abdominal distension, and constipation (10.6% vs. 5.6%) occurring more frequently in the OCT-LAR arm compared to the PAS-LAR arm [38]. However, other studies have reported different results, with diarrhea occurring in 22.2% of patients receiving PAS-LAR and 18.4% of those receiving OCT-LAR, regardless of whether there is a suspected relationship to the study drug [71].

An increased risk of biliary adverse events, including cholelithiasis, is common with all SRLs [72], and along with diarrhea, it is one of the most frequent ADRs associated with PAS therapy in acromegaly, following hyperglycemia-related events. New gallstones have been reported in approximately 15% of patients undergoing long-acting analog therapy, typically within the first year of treatment. Additionally, some patients may develop gallbladder sludge or microlithiasis [5]. Gallstones may cause acute biliary complications such as pancreatitis, cholecystitis, or biliary colic. Most patients, however, remain asymptomatic. Similar frequencies of signals were reported for all drugs in the “Hepatobiliary disorders” SOC. However, the disproportionality analysis indicated a higher likelihood of reporting hepatobiliary stones with PAS compared to OCT, PEG, and all other drugs, except LAN. Interestingly, clinical studies have shown the opposite results, with cholelithiasis occurring more frequently with OCT-LAR (36–39.4%) than with PAS-LAR (26–30%) [20,37,38], but not always [71]. In a long-term real-life study of acromegaly patients treated with PAS, 6 out of 29 patients (21%) who had normal baseline ultrasounds developed biliary sludge, and 3 patients (10%) developed cholelithiasis over 11 years of treatment. However, given the prolonged nature of the treatment, this outcome was not unexpected [34]. One of the most commonly reported side effects of PEG is elevated liver transaminases, with an overall incidence of 3.0% [73]. While no cases of liver failure have been documented in the current literature, two cases of hepatitis associated with PEG treatment have been reported [74,75].

### Limitations of this Study

To provide a fair assessment and interpretation of the data analyzed, several limitations of this study should be addressed. The issues of underreporting, overreporting, and reporting bias are frequently encountered in spontaneous reporting systems, and they can lead to incomplete and inaccurate data provided in ICSRs; thus, information regarding clinical characteristics, comorbidities, other administered drugs, and outcomes may be lacking. Other factors that can influence the number of reported ADRs could be the usage extent of the analyzed drug, the severity of the outcome, and the reporting variability between different regions, with emphasis on the ones with limited healthcare infrastructure. The descriptive and disproportionality analyses performed in this study were based on the identification of a safety signal; thus, the data provided should not be used alone when performing safety profile analysis for SRLs. Moreover, further studies should be performed to assess whether there is a causal relationship between the use of SRLs and the reported ADRs and to provide a complete and accurate safety profile of the analyzed drugs.

## 4. Material and Methods

A pharmacovigilance study on ADRs reported for PAS was conducted using ICSRs submitted to the EV database (https://www.adrreports.eu/) on 11 August 2024. Initially, a descriptive analysis of all cases reported in EV was performed [76]. This included a comparison between PAS and other drugs used in acromegaly management (OCT, LAN, and PEG) focusing on the total number of ICSRs, the total ADRs reported for each drug, and the general characteristics recorded in the ICSRs: patients’ age groupss (0–1 month, 2 months–2 years, 3–11 years, 12–17 years, 18–64 years, 65–85 years, over 85 years, or not specified (NS)), sex (male, female, or NS), geographic origin (EEA, non-EEA, or NS), and reporter category (HP, non-HP, or NS). Furthermore, the distribution of cases by seriousness (serious, non-serious, or NS) was compared across PAS and OCT, LAN, and PEG. An adverse reaction was considered serious if it resulted in death, persistent or significant disability or incapacity, or a birth defect; was life-threatening; or required hospitalization or prolongation of existing hospitalization [77]. For PAS, the distribution of serious cases was further analyzed for each SOC.

In pharmacovigilance studies, the probability of ADR reporting is assessed through disproportionality analysis. According to the EMA recommendations, the ROR and 95% confidence intervals (CI) should be calculated in order to identify similarities and differences in ADR reporting. ROR compares cases reported for the drug of interest with those for all other drugs in the database or with drugs used in related therapeutic areas or similar clinical contexts [78,79].

The analysis is valid when there are over five cases for each ADR, and the lower limit of the 95% CI is above 1 [80,81]. The equations used for calculating each parameter are presented below [80,81]:

$$ROR=\frac{a\;x\;d\;}{b\;x\;c}$$

where the equation elements are defined as follows:

- *ROR* = reporting odds ratio;
- *a* = evaluated ADR for targeted drug;
- *b* = other ADRs for targeted drug;
- *c* = evaluated ADR for drug used for comparison;
- *d* = other ADRs for drug used for comparison.

95% CI = exp (ln (*ROR*) − 1.96 × SE{ln(*ROR*)}) to exp (ln(*ROR*) + 1.96 × SE{ln(*ROR*)})

where the equation elements are defined as follows:

- CI = confidence interval;
- SE = standard error.

$$SE\{ln\left(ROR\right)\}=\sqrt{\frac{1}{a}+\frac{1}{b}+\frac{1}{c}+\frac{1}{d}}$$

The ROR and 95% CI were calculated using MedCalc Software Ltd. The odds ration was determined using the odds ratio calculator on https://www.medcalc.org/calc/odds_ratio.php (Version 23.0.9) accessed on 15 October 2024 [82].

To evaluate PAS risk, a disproportionality analysis was conducted. Based on findings from the scientific literature, the following SOCs were chosen to evaluate the safety profile of PAS compared to other acromegaly treatments: “Cardiac disorders”, “Endocrine disorders”, “Gastrointestinal disorders”, “Hepatobiliary disorders”, “Metabolism and nutrition disorders”, and “Vascular disorders”. For further analysis, 82 Preferred Terms (PTs) within these SOCs and related to various high-impact medical conditions affecting patients’ quality of life were selected (Table 2). All data retrieved from the EV platform were anonymized, and no ethical approval was required for conducting the descriptive or disproportionality analyses.

## 5. Conclusions

Acromegaly is associated with a significant treatment burden and high mortality rates, primarily due to malignancies and cardiovascular and respiratory disorders. Since patients often receive delayed diagnoses, surgery may be insufficient or inappropriate, making long-term medical therapy necessary. The evaluation of real-world data from the EV database on the safety profile of PAS revealed that it had the fewest ICSRs and ADRs compared to LAN, PEG, and especially OCT. Serious ADRs were less frequent with PAS and LAN than with PEG and OCT. In the “Gastrointestinal Disorders” category, PAS showed fewer reports of abdominal pain and defecation disorders compared to the fg-SRLs OCT and LAN, with fewer serious ADRs in this category. The frequency of hepatobiliary disorders was similar for all drugs, though PAS had a higher likelihood of causing hepatobiliary stones than OCT and PEG. Cardiovascular ADRs were the least common with PAS, but PAS had more reports in the “Metabolism and Nutrition Disorders” and ”Endocrine disorders” categories compared to other SRLs and PEG. Moreover, these events are frequently classified as serious. These included events like disturbances in carbohydrate metabolism, elevated blood glucose levels, IGT, or diabetes, which are expected given PAS’s effects on pancreatic islet cells and incretin release. Overall, PAS is generally well tolerated, with the most common adverse events being metabolic disturbances and the development of hepatobiliary stones. Therefore, regular blood glucose monitoring is essential, particularly for patients with pre-existing diabetes or those at risk. Additionally, since gallbladder-related issues, particularly gallstones, may occur, patients treated with PAS require monitoring through ultrasound and liver function tests. Regular assessments are crucial for the timely identification and management of these potential complications.

## Figures and Tables

**Figure 8:**
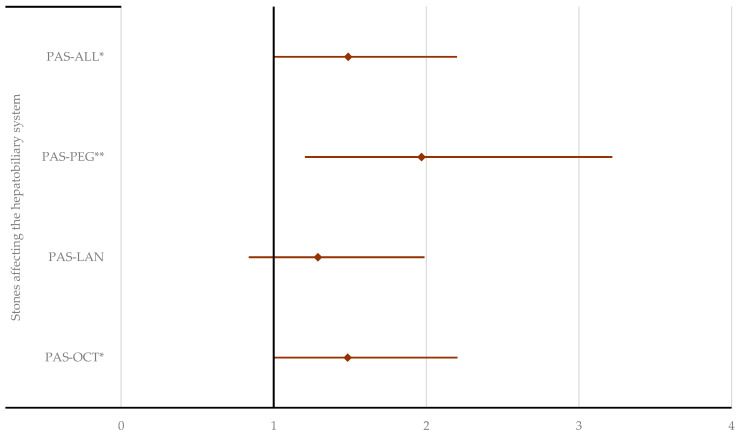
Disproportionality analysis for cholelithiasis. ALL—all drugs except pasireotide; LAN—lanreotide; OCT—octreotide; PAS—pasireotide; PEG—pegvisomant. * *p* < 0.05; ** *p* ≤ 0.01.

**Table 1:** Characteristics of ICSRs reported in EV for acromegaly drugs. EEA—European Economic Area; HP—healthcare professional; LAN—lanreotide; OCT—octreotide; PAS—pasireotide; PEG—pegvisomant.

	PAS	OCT	LAN	PEG
	*n*	*n*	*n*	*n*
	(%)	(%)	(%)	(%)
Total	698	10,613	3012	1765
	(100%)	(100%)	(100%)	(100%)
Age category				
Not Specified	199	4285	971	265
	(28.51%)	(40.38%)	(32.24%)	(15.01%)
0–1 Month	3	58	2	0
	(0.43%)	(0.55%)	(0.07%)	(0.00%)
2 Months–2 Years	0	70	12	0
	(0.00%)	(0.66%)	(0.40%)	(0.00%)
3–11 Years	2	71	11	6
	(0.29%)	(0.67%)	(0.37%)	(0.34%)
12–17 Years	3	56	12	18
	(0.43%)	(0.53%)	(0.40%)	(1.02%)
18–64 Years	387	3574	1170	1024
	(55.44%)	(33.68%)	(38.84%)	(58.02%)
65–85 Years	103	2348	786	432
	(14.76%)	(22.12%)	(26.1%)	(24.48%)
More than 85 Years	1	151	48	20
	(0.14%)	(1.42%)	(1.59%)	(1.13%)
Sex				
Female	435	5269	1573	1012
	(62.32%)	(49.65%)	(52.22%)	(57.34%)
Male	202	4442	1317	700
	(28.94%)	(41.85%)	(43.73%)	(39.66%)
Not Specified	61	902	122	53
	(8.74%)	(8.50%)	(4.05%)	(3.00%)
Geographic origin				
EEA	393	3186	1801	812
	(56.3%)	(30.02%)	(59.79%)	(46.01%)
NON-EEA	305	7427	1211	953
	(43.7%)	(69.98%)	(40.21%)	(53.99%)
Not Specified	0	10,613	0	0
	(0.00%)	(100.00%)	(0.00%)	(0.00%)
Reporter				
HP	578	9136	2284	1223
	(82.81%)	(86.08%)	(75.83%)	(69.29%)
Non HP	120	1436	728	542
	(17.19%)	(13.53%)	(24.17%)	(30.71%)
Not Specified	0	41	0	0
	(0.00%)	(0.39%)	(0.00%)	(0.00%)

**Table 2:** PTs selected for disproportionality analysis.

SOC	Medical Condition	PT
Gastrointestinal disorders	Abdominal distress	Abdominal discomfort
		Abdominal symptom
		Abdominal pain
		Abdominal pain lower
		Abdominal pain upper
		Abdominal tenderness
	Abnormalities in stool appearance and consistency	Abnormal feces
		Feces discolored
		Feces pale
		Feces hard
		Feces soft
		Steatorrhea
	Bowel movement disorders	Defecation disorder
		Defecation urgency
		Diarrhea
		Constipation
		Change in bowel habits
		Bowel movement irregularity
		Frequent bowel movements
	Pancreatitis (including acute and chronic)	Pancreatitis
		Pancreatitis acute
		Pancreatitis chronic
	Vomiting	Vomiting
	Pancreatolithiasis *	Pancreatolithiasis
	Proctalgia *	Proctalgia
	Eructation *	Eructation
	Flatulence	Flatulence
	Acute abdomen *	Acute abdomen
	Gastric hemorrhage *	Gastric hemorrhage
	Gastric perforation *	Gastric perforation
	Hematemesis *	Hematemesis
Hepatobiliary disorders	Stones affecting the hepatobiliary system	Bile duct stone
		Cholelithiasis
	Cholestatic conditions *	Cholestasis
		Jaundice cholestatic
	Jaundice *	Jaundice
	Hepatic failure (including acute hepatic failure) *	Acute hepatic failure
		Hepatic failure
	Hepatitis acute *	Hepatitis acute
	Biliary obstruction *	Biliary obstruction
	Cholangitis/cholangitis acute *	Cholangitis
		Cholangitis acute
	Biliary obstruction *	Hepatorenal syndrome
Cardiac disorders	Cardiac arrhythmias	Arrhythmia
		Atrial fibrillation
		Atrioventricular block complete
		Bradycardia
	Cardiac failure (including cardiac failure congestive) *	Cardiac failure
		Cardiac failure congestive
	Acute myocardial infarction *	Acute myocardial infarction
	Cardiac arrest (including cardio-respiratory arrest) *	Cardiac arrest
		Cardio-respiratory arrest
	Cardiogenic shock *	Cardiogenic shock
	Cardiopulmonary failure *	Cardiopulmonary failure
Vascular	Hypertension *	Hypertension
	Hypotension	Hypotension
	Hemorrhage *	Hemorrhage
	Embolism *	Embolism
	Thrombotic events *	Thrombosis
		Deep vein thrombosis
	Hypertensive crisis *	Hypertensive crisis
	Hypovolemic shock *	Hypovolemic shock
	Neurogenic shock *	Neurogenic shock
	Circulatory collapse *	Circulatory collapse
Endocrine disorders	Adrenal insufficiency	Adrenal insufficiency
	Hypopituitarism *	Hypopituitarism
	Thyroid dysfunctions *	Hypothyroidism
		Hyperthyroidism
Metabolism and nutrition disorders	Electrolyte disorders	Electrolyte imbalance
		Hypercalcemia
		Hypocalcemia
		Hypokalemia
		Hyponatremia
	Hypoalbuminemia *	Hypoalbuminemia
	Hypercholesterolemia *	Hypercholesterolemia
	Gout *	Gout
	Hypoglycemia	Hypoglycemia
	Glucose tolerance impaired	Glucose tolerance impaired
	Diabetes mellitus	Diabetes mellitus
	Type 1 diabetes *	Type 1 diabetes
	Type 2 diabetes	Type 2 diabetes
	Diabetic Ketoacidosis	Diabetic Ketoacidosis

* The disproportionality could not be established because the number of cases reported for PAS was fewer than 5.

## Data Availability

The data are contained within the article.

## Supplementary Materials

The following supporting information can be downloaded at: https://www.mdpi.com/article/10.3390/ph17121631/s1, Table S1: Distribution of ADRs by SOC for drugs used in the treatment of acromegaly. Table S2: Number of ADRs related to selected SOCs. ALL—all other drugs excepted pasireotide; Table S3: Number of ADRs reported for gastro-intestinal ADRs. ALL—all other drugs excepted pasireotide; Table S4: Number of ADRs reported for cholelithiasis. ALL—all other drugs excepted pasireotide; Table S5: Number of ADRs reported for hypotension. ALL—all other drugs excepted pasireotide; Table S6: Number of ADRs reported for hypotension. ALL—all other drugs excepted pasireotide; Table S7: Number of ADRs reported for metabolic and nutritional disorders.

## References

[1] Buttan A., Mamelak A.N. (2019). Endocrine Outcomes After Pituitary Surgery. Neurosurg. Clin. N. Am..

[2] Mortini P., Barzaghi L.R., Albano L., Panni P., Losa M. (2018). Microsurgical Therapy of Pituitary Adenomas. Endocrine.

[3] Fleseriu M., Biller B.M.K., Freda P.U., Gadelha M.R., Giustina A., Katznelson L., Molitch M.E., Samson S.L., Strasburger C.J., van der Lely A.J. (2021). A Pituitary Society Update to Acromegaly Management Guidelines. Pituitary.

[4] Giustina A., Biermasz N., Casanueva F.F., Fleseriu M., Mortini P., Strasburger C., van der Lely A.J., Wass J., Melmed S., Banfi G. (2023). Consensus on Criteria for Acromegaly Diagnosis and Remission. Pituitary.

[5] Freda P.U. (2002). Somatostatin Analogs in Acromegaly. J. Clin. Endocrinol. Metab..

[6] Günther T., Tulipano G., Dournaud P., Bousquet C., Csaba Z., Kreienkamp H.-J., Lupp A., Korbonits M., Castaño J.P., Wester H.-J. (2018). International Union of Basic and Clinical Pharmacology. CV. Somatostatin Receptors: Structure, Function, Ligands, and New Nomenclature. Pharmacol. Rev..

[7] Melmed S. (2016). New Therapeutic Agents for Acromegaly. Nat. Rev. Endocrinol..

[8] Reubi J., Waser B., Schaer J.-C., Laissue J.A. (2001). Somatostatin Receptor Sst1–Sst5 Expression in Normal and Neoplastic Human Tissues Using Receptor Autoradiography with Subtype-Selective Ligands. Eur. J. Nucl. Med..

[9] Hofland L.J., Feelders R.A., de Herder W.W., Lamberts S.W.J. (2010). Pituitary Tumours: The Sst/D2 Receptors as Molecular Targets. Mol. Cell. Endocrinol..

[10] Fleseriu M., Rusch E., Geer E.B. (2017). Safety and Tolerability of Pasireotide Long-Acting Release in Acromegaly—Results from the Acromegaly, Open-Label, Multicenter, Safety Monitoring Program for Treating Patients Who Have a Need to Receive Medical Therapy (ACCESS) Study. Endocrine.

[11] Bolanowski M., Kałużny M., Witek P., Jawiarczyk-Przybyłowska A. (2022). Pasireotide—A Novel Somatostatin Receptor Ligand after 20 Years of Use. Rev. Endocr. Metab. Disord..

[12] Fleseriu M., Cuevas-Ramos D. (2016). Pasireotide: A Novel Treatment for Patients with Acromegaly. Drug Des. Dev. Ther..

[13] Colao A., Petersenn S., Newell-Price J., Findling J.W., Gu F., Maldonado M., Schoenherr U., Mills D., Salgado L.R., Biller B.M.K. (2012). A 12-Month Phase 3 Study of Pasireotide in Cushing’s Disease. N. Engl. J. Med..

[14] van der Hoek J., Waaijers M., van Koetsveld P.M., Sprij-Mooij D., Feelders R.A., Schmid H.A., Schoeffter P., Hoyer D., Cervia D., Taylor J.E. (2005). Distinct Functional Properties of Native Somatostatin Receptor Subtype 5 Compared with Subtype 2 in the Regulation of ACTH Release by Corticotroph Tumor Cells. Am. J. Physiol.-Endocrinol. Metab..

[15] Hofland L.J., van der Hoek J., Feelders R., van Aken M.O., van Koetsveld P.M., Waaijers M., Sprij-Mooij D., Bruns C., Weckbecker G., de Herder W.W. (2005). The Multi-Ligand Somatostatin Analogue SOM230 Inhibits ACTH Secretion by Cultured Human Corticotroph Adenomas via Somatostatin Receptor Type 5. Eur. J. Endocrinol..

[16] Batista D.L., Zhang X., Gejman R., Ansell P.J., Zhou Y., Johnson S.A., Swearingen B., Hedley-Whyte E.T., Stratakis C.A., Klibanski A. (2006). The Effects of SOM230 on Cell Proliferation and Adrenocorticotropin Secretion in Human Corticotroph Pituitary Adenomas. J. Clin. Endocrinol. Metab..

[17] Lesche S., Lehmann D., Nagel F., Schmid H.A., Schulz S. (2009). Differential Effects of Octreotide and Pasireotide on Somatostatin Receptor Internalization and Trafficking in Vitro. J. Clin. Endocrinol. Metab..

[18] Bruns C., Lewis I., Briner U., Meno-Tetang G., Weckbecker G. (2002). SOM230: A Novel Somatostatin Peptidomimetic with Broad Somatotropin Release Inhibiting Factor (SRIF) Receptor Binding and a Unique Antisecretory Profile. Eur. J. Endocrinol..

[19] Lacroix A., Gu F., Gallardo W., Pivonello R., Yu Y., Witek P., Boscaro M., Salvatori R., Yamada M., Tauchmanova L. (2018). Efficacy and Safety of Once-Monthly Pasireotide in Cushing’s Disease: A 12 Month Clinical Trial. Lancet Diabetes Endocrinol..

[20] Colao A., Bronstein M.D., Freda P., Gu F., Shen C.-C., Gadelha M., Fleseriu M., van der Lely A.J., Farrall A.J., Hermosillo Reséndiz K. (2014). Pasireotide versus Octreotide in Acromegaly: A Head-to-Head Superiority Study. J. Clin. Endocrinol. Metab..

[21] Giustina A., Barkhoudarian G., Beckers A., Ben-Shlomo A., Biermasz N., Biller B., Boguszewski C., Bolanowski M., Bollerslev J., Bonert V. (2020). Multidisciplinary Management of Acromegaly: A Consensus. Rev. Endocr. Metab. Disord..

[22] Lamberts S.W.J., Uitterlinden P., Verschoor L., van Dongen K.J., del Pozo E. (1985). Long-Term Treatment of Acromegaly with the Somatostatin Analogue SMS 201–995. N. Engl. J. Med..

[23] van der Lely A.J., Hutson R.K., Trainer P.J., Besser G.M., Barkan A.L., Katznelson L., Klibanski A., Herman-Bonert V., Melmed S., Vance M.L. (2001). Long-Term Treatment of Acromegaly with Pegvisomant, a Growth Hormone Receptor Antagonist. Lancet.

[24] van der Lely A.J., Biller B.M.K., Brue T., Buchfelder M., Ghigo E., Gomez R., Hey-Hadavi J., Lundgren F., Rajicic N., Strasburger C.J. (2012). Long-Term Safety of Pegvisomant in Patients with Acromegaly: Comprehensive Review of 1288 Subjects in ACROSTUDY. J. Clin. Endocrinol. Metab..

[25] Haberbosch L., Strasburger C.J. (2023). Efficacy and Safety of Pegvisomant in the Treatment of Acromegaly. Arch. Med. Res..

[26] EudraVigilance European Database of Suspected Adverse Drug Reaction Reports. https://www.adrreports.eu/ro/eudravigilance.html.

[27] Akirov A., Gorshtein A., Dotan I., Khazen N.S., Pauker Y., Gershinsky M., Shimon I. (2021). Long-Term Safety and Efficacy of Long-Acting Pasireotide in Acromegaly. Endocrine.

[28] Witek P., Bolanowski M., Szamotulska K., Wojciechowska-Luźniak A., Jawiarczyk-Przybyłowska A., Kałużny M. (2021). The Effect of 6 Months’ Treatment with Pasireotide LAR on Glucose Metabolism in Patients with Resistant Acromegaly in Real-World Clinical Settings. Front. Endocrinol..

[29] Stelmachowska-Banaś M., Czajka-Oraniec I., Tomasik A., Zgliczyński W. (2022). Real-World Experience with Pasireotide-LAR in Resistant Acromegaly: A Single Center 1-Year Observation. Pituitary.

[30] Lasolle H., Ferriere A., Vasiljevic A., Eimer S., Nunes M.-L., Tabarin A. (2019). Pasireotide-LAR in Acromegaly Patients Treated with a Combination Therapy: A Real-Life Study. Endocr. Connect..

[31] Shimon I., Adnan Z., Gorshtein A., Baraf L., Saba Khazen N., Gershinsky M., Pauker Y., Abid A., Niven M.J., Shechner C. (2018). Efficacy and Safety of Long-Acting Pasireotide in Patients with Somatostatin-Resistant Acromegaly: A Multicenter Study. Endocrine.

[32] Coopmans E.C., Muhammad A., van der Lely A.J., Janssen J.A.M.J.L., Neggers S.J.C.M.M. (2019). How to Position Pasireotide LAR Treatment in Acromegaly. J. Clin. Endocrinol. Metab..

[33] Daly A.F., Potorac I., Petrossians P., Beckers A. (2019). Shrinkage of Pituitary Adenomas with Pasireotide. Lancet Diabetes Endocrinol..

[34] Gadelha M., Marques N.V., Fialho C., Scaf C., Lamback E., Antunes X., Santos E., Magalhães J., Wildemberg L.E. (2023). Long-Term Efficacy and Safety of Pasireotide in Patients with Acromegaly: 14 Years of Single-Center Real-World Experience. J. Clin. Endocrinol. Metab..

[35] Recordati (2020). Signifor LAR Prescribing Information. https://www.accessdata.fda.gov/drugsatfda_docs/label/2020/203255s008lbl.pdf.

[36] Colao A., Bronstein M.D., Brue T., De Marinis L., Fleseriu M., Guitelman M., Raverot G., Shimon I., Fleck J., Gupta P. (2020). Pasireotide for Acromegaly: Long-Term Outcomes from an Extension to the Phase III PAOLA Study. Eur. J. Endocrinol..

[37] Gadelha M.R., Bronstein M.D., Brue T., Coculescu M., Fleseriu M., Guitelman M., Pronin V., Raverot G., Shimon I., Lievre K.K. (2014). Pasireotide versus Continued Treatment with Octreotide or Lanreotide in Patients with Inadequately Controlled Acromegaly (PAOLA): A Randomised, Phase 3 Trial. Lancet Diabetes Endocrinol..

[38] Sheppard M., Bronstein M.D., Freda P., Serri O., De Marinis L., Naves L., Rozhinskaya L., Hermosillo Reséndiz K., Ruffin M., Chen Y. (2015). Pasireotide LAR Maintains Inhibition of GH and IGF-1 in Patients with Acromegaly for up to 25 Months: Results from the Blinded Extension Phase of a Randomized, Double-Blind, Multicenter, Phase III Study. Pituitary.

[39] Biagetti B., Araujo-Castro M., Tebe C., Marazuela M., Puig-Domingo M. (2024). Real-World Evidence of Effectiveness and Safety of Pasireotide in the Treatment of Acromegaly: A Systematic Review and Meta-Analysis. Rev. Endocr. Metab. Disord..

[40] Aliyeva T., Muniz J., Soares G.M., Firdausa S., Mirza L. (2024). Efficacy and Safety of Pasireotide Treatment in Acromegaly: A Systematic Review and Single Arm Meta-Analysis. Pituitary.

[41] Astruc B., Marbach P., Bouterfa H., Denot C., Safari M., Vitaliti A., Sheppard M. (2005). Long-Acting Octreotide and Prolonged-Release Lanreotide Formulations Have Different Pharmacokinetic Profiles. J. Clin. Pharmacol..

[42] Bornschein J., Drozdov I., Malfertheiner P. (2009). Octreotide LAR: Safety and Tolerability Issues. Expert Opin. Drug Saf..

[43] Chanson P., Boerlin V., Ajzenberg C., Bachelot Y., Benito P., Bringer J., Caron P., Charbonnel B., Cortet C., Delemer B. (2000). Comparison of Octreotide Acetate LAR and Lanreotide SR in Patients with Acromegaly. Clin. Endocrinol..

[44] Kasuki L., Wildemberg L.E., Gadelha M.R. (2018). Management of endocrine disease: Personalized Medicine in the Treatment of Acromegaly. Eur. J. Endocrinol..

[45] Petersenn S., Houchard A., Sert C., Caron P.J., PRIMARYS Study Group (2020). Predictive Factors for Responses to Primary Medical Treatment with Lanreotide Autogel 120 Mg in Acromegaly: Post Hoc Analyses from the PRIMARYS Study. Pituitary.

[46] Berton A.M., Prencipe N., Bertero L., Baldi M., Bima C., Corsico M., Bianchi A., Mantovani G., Ferraù F., Sartorato P. (2022). Resistance to Somatostatin Analogs in Italian Acromegaly Patients: The MISS Study. J. Clin. Med..

[47] Newell-Price J., Bertagna X., Grossman A.B., Nieman L.K. (2006). Cushing’s Syndrome. Lancet.

[48] Nakagawa K., Kajiwara A. (2015). Female Sex as a Risk Factor for Adverse Drug Reactions. Nihon Rinsho.

[49] Tharpe N. (2011). Adverse Drug Reactions in Women’s Health Care. J. Midwifery Womens Health.

[50] Lenders N.F., McCormack A.I., Ho K.K.Y. (2020). Management of endocrine disease: Does Gender Matter in the Management of Acromegaly?. Eur. J. Endocrinol..

[51] Dal J., Feldt-Rasmussen U., Andersen M., Kristensen L.Ø., Laurberg P., Pedersen L., Dekkers O.M., Sørensen H.T., Jørgensen J.O.L. (2016). Acromegaly Incidence, Prevalence, Complications and Long-Term Prognosis: A Nationwide Cohort Study. Eur. J. Endocrinol..

[52] Novacek G. (2006). Gender and Gallstone Disease. Wien. Med. Wochenschr..

[53] Smith W.H.T., Nair R.U., Adamson D., Kearney M.T., Ball S.G., Balmforth A.J. (2005). Somatostatin Receptor Subtype Expression in the Human Heart: Differential Expression by Myocytes and Fibroblasts. J. Endocrinol..

[54] Pasireotide: Drug information. https://medilib.ir/uptodate/show/87457.

[55] Breitschaft A., Hu K., Darstein C., Ligueros-Saylan M., Jordaan P., Song D., Hudson M., Shah R. (2014). Effects of Subcutaneous Pasireotide on Cardiac Repolarization in Healthy Volunteers: A Single-center, Phase I, Randomized, Four-way Crossover Study. J. Clin. Pharmacol..

[56] Lombardi G., Colao A., Ferone D., Marzullo P., Landi M.L., Longobardi S., Iervolino E., Cuocolo A., Fazio S., Merola B. (1996). Cardiovascular Aspects in Acromegaly: Effects of Treatment. Metabolism.

[57] Fatti L.M., Scacchi M., Lavezzi E., Giraldi F.P., De Martin M., Toja P., Michailidis G., Stramba-Badiale M., Cavagnini F. (2006). Effects of Treatment with Somatostatin Analogues on QT Interval Duration in Acromegalic Patients. Clin. Endocrinol..

[58] Pecori Giraldi F., Toja P.M., Michailidis G., Metinidou A., De Martin M., Scacchi M., Stramba-Badiale M., Cavagnini F. (2011). High Prevalence of Prolonged QT Interval Duration in Male Patients with Cushing’s Disease. Exp. Clin. Endocrinol. Diabetes.

[59] Bubiński R., Kuś W., Goch J. (1993). Effect of Somatostatin on the Conduction System of the Heart. Kardiol. Pol..

[60] van der Pas R., de Bruin C., Leebeek F.W.G., de Maat M.P.M., Rijken D.C., Pereira A.M., Romijn J.A., Netea-Maier R.T., Hermus A.R., Zelissen P.M.J. (2012). The Hypercoagulable State in Cushing’s Disease Is Associated with Increased Levels of Procoagulant Factors and Impaired Fibrinolysis, But Is Not Reversible after Short-Term Biochemical Remission Induced by Medical Therapy. J. Clin. Endocrinol. Metab..

[61] Somavert-Summary of Product Characteristics. https://ec.europa.eu/health/documents/community-register/2009/2009112070108/anx_70108_en.pdf.

[62] Kuhn E., Maione L., Bouchachi A., Rozière M., Salenave S., Brailly-Tabard S., Young J., Kamenicky P., Assayag P., Chanson P. (2015). Long-Term Effects of Pegvisomant on Comorbidities in Patients with Acromegaly: A Retrospective Single-Center Study. Eur. J. Endocrinol..

[63] Giustina A., Barkan A., Beckers A., Biermasz N., Biller B.M.K., Boguszewski C., Bolanowski M., Bonert V., Bronstein M.D., Casanueva F.F. (2020). A Consensus on the Diagnosis and Treatment of Acromegaly Comorbidities: An Update. J. Clin. Endocrinol. Metab..

[64] Maione L., Brue T., Beckers A., Delemer B., Petrossians P., Borson-Chazot F., Chabre O., François P., Bertherat J., Cortet-Rudelli C. (2017). Changes in the Management and Comorbidities of Acromegaly over Three Decades: The French Acromegaly Registry. Eur. J. Endocrinol..

[65] Henry R.R., Ciaraldi T.P., Armstrong D., Burke P., Ligueros-Saylan M., Mudaliar S. (2013). Hyperglycemia Associated with Pasireotide: Results From a Mechanistic Study in Healthy Volunteers. J. Clin. Endocrinol. Metab..

[66] Cuevas-Ramos D., Fleseriu M. (2014). Somatostatin Receptor Ligands and Resistance to Treatment in Pituitary Adenomas. J. Mol. Endocrinol..

[67] Godara A., Siddiqui N.S., Byrne M.M., Saif M.W. (2018). The safety of lanreotide for neuroendocrine tumor. Expert Opin. Drug Saf..

[68] Fleseriu M., Iweha C., Salgado L., Mazzuco T.L., Campigotto F., Maamari R., Limumpornpetch P. (2019). Safety and Efficacy of Subcutaneous Pasireotide in Patients With Cushing’s Disease: Results From an Open-Label, Multicenter, Single-Arm, Multinational, Expanded-Access Study. Front. Endocrinol..

[69] Petersenn S., Schopohl J., Barkan A., Mohideen P., Colao A., Abs R., Buchelt A., Ho Y.-Y., Hu K., Farrall A.J. (2010). Pasireotide (SOM230) Demonstrates Efficacy and Safety in Patients with Acromegaly: A Randomized, Multicenter, Phase II Trial. J. Clin. Endocrinol. Metab..

[70] Petersenn S., Farrall A.J., Block C., Melmed S., Schopohl J., Caron P., Cuneo R., Kleinberg D., Colao A., Ruffin M. (2014). Long-Term Efficacy and Safety of Subcutaneous Pasireotide in Acromegaly: Results from an Open-Ended, Multicenter, Phase II Extension Study. Pituitary.

[71] Bronstein M.D., Fleseriu M., Neggers S., Colao A., Sheppard M., Gu F., Shen C.-C., Gadelha M., Farrall A.J., Hermosillo Reséndiz K. (2016). Switching Patients with Acromegaly from Octreotide to Pasireotide Improves Biochemical Control: Crossover Extension to a Randomized, Double-Blind, Phase III Study. BMC Endocr. Disord..

[72] Prencipe N., Bona C., Cuboni D., Parasiliti-Caprino M., Berton A.M., Fenoglio L.M., Gasco V., Ghigo E., Grottoli S. (2021). Biliary Adverse Events in Acromegaly during Somatostatin Receptor Ligands: Predictors of Onset and Response to Ursodeoxycholic Acid Treatment. Pituitary.

[73] Leonart L.P., Tonin F.S., Ferreira V.L., Fernandez-Llimos F., Pontarolo R. (2019). Effectiveness and Safety of Pegvisomant: A Systematic Review and Meta-Analysis of Observational Longitudinal Studies. Endocrine.

[74] Biering H., Saller B., Bauditz J., Pirlich M., Rudolph B., Johne A., Buchfelder M., Mann K., Droste M., Schreiber I. (2006). Elevated Transaminases during Medical Treatment of Acromegaly: A Review of the German Pegvisomant Surveillance Experience and a Report of a Patient with Histologically Proven Chronic Mild Active Hepatitis. Eur. J. Endocrinol..

[75] Feenstra J., van Aken M.O., de Herder W.W., Feelders R.A., van der Lely A.J. (2006). Drug-Induced Hepatitis in an Acromegalic Patient during Combined Treatment with Pegvisomant and Octreotide Long-Acting Repeatable Attributed to the Use of Pegvisomant. Eur. J. Endocrinol..

[76] Online Access to Suspected Side-Effect Reports. https://www.adrreports.eu/ro/disclaimer.html.

[77] European Medicines Agency Serious Adverse Reaction. https://www.ema.europa.eu/en/glossary-terms/serious-adverse-reaction.

[78] Morgovan C., Dobrea C.M., Chis A.A., Juncan A.M., Arseniu A.M., Rus L.L., Gligor F.G., Ardelean S.A., Stoicescu L., Ghibu S. (2023). A Descriptive Analysis of Direct Oral Anticoagulant Drugs Dosing Errors Based on Spontaneous Reports from the EudraVigilance Database. Pharmaceuticals.

[79] Grundmark B., Holmberg L., Garmo H., Zethelius B. (2014). Reducing the Noise in Signal Detection of Adverse Drug Reactions by Standardizing the Background: A Pilot Study on Analyses of Proportional Reporting Ratios-by-Therapeutic Area. Eur. J. Clin. Pharmacol..

[80] Pop G., Farcaș A., Butucă A., Morgovan C., Arseniu A.M., Pumnea M., Teodoru M., Gligor F.G. (2022). Post-Marketing Surveillance of Statins—A Descriptive Analysis of Psychiatric Adverse Reactions in EudraVigilance. Pharmaceuticals.

[81] European Medicine Agency Screening for Adverse Reactions in EudraVigilance. https://www.ema.europa.eu/en/documents/other/screening-adverse-reactions-eudravigilance_en.pdf.

[82] https://www.medcalc.org/calc/odds_ratio.php.

