# Supplementary material for: Real-Life Data on the Safety of Pasireotide in Acromegaly: Insights from EudraVigilance
Source: Pharmaceuticals (Basel). 2024 Dec 4;17(12):1631. doi: 10.3390/ph17121631 (PMC11728653; doi:10.3390/ph17121631)
Supplement: Supplementary file 1 [file pharmaceuticals-17-01631-s001.zip › pharmaceuticals-3308733-supplementary.pdf]

Table S1. Distribution of ADRs by SOC for drugs used in the treatment of acromegaly.

	**PASIREOTIDE**	**OCTREOTIDE**	**LANREOTIDE**	**PEGVISOMANT**
Blood and lymphatic system disorders	1.1%	1.4%	1.2%	0.5%
Cardiac disorders	1.9%	2.9%	2.7%	2.4%
Congenital, familial and genetic disorders	0.1%	0.2%	0.1%	0.4%
Ear and labyrinth disorders	0.2%	0.5%	0.3%	0.5%
Endocrine disorders	3.2%	1.3%	1.4%	1.7%
Eye disorders	1.9%	1.1%	1.0%	1.7%
Gastrointestinal disorders	10.0%	10.7%	13.3%	6.7%
General disorders and administration site conditions	14.3%	15.0%	18.5%	16.3%
Hepatobiliary disorders	3.0%	3.2%	3.1%	2.8%
Immune system disorders	0.2%	0.9%	0.5%	1.2%
Infections and infestations	2.6%	4.0%	3.8%	4.5%
Injury, poisoning and procedural complications	8.1%	6.2%	10.7%	12.6%
Investigations	11.7%	8.9%	5.8%	9.2%
Metabolism and nutrition disorders	17.5%	4.6%	4.5%	2.7%
Musculoskeletal and connective tissue disorders	2.2%	4.2%	3.5%	5.5%
Neoplasms benign, malignant and unspecified (incl cysts and polyps)	4.1%	8.5%	5.5%	6.0%
Nervous system disorders	5.2%	5.9%	5.8%	7.6%
Pregnancy, puerperium and perinatal conditions	0.4%	0.3%	0.1%	0.2%
Product issues	0.8%	2.5%	2.0%	2.4%
Psychiatric disorders	1.3%	2.9%	2.2%	2.9%
Renal and urinary disorders	2.5%	1.9%	1.6%	1.5%
Reproductive system and breast disorders	0.8%	0.6%	0.5%	0.4%
Respiratory, thoracic and mediastinal disorders	1.5%	3.8%	2.1%	2.7%
Skin and subcutaneous tissue disorders	2.9%	3.5%	4.1%	3.7%
Social circumstances	0.0%	0.1%	0.3%	0.4%
Surgical and medical procedures	0.5%	1.2%	2.1%	1.2%
Vascular disorders	1.8%	3.8%	3.3%	2.4%

Table S2 - Number of ADRs related to selected SOCs. ALL – all other drugs excepted pasireotide; LAN – lanreotide, OCT – octreotide; PAS - pasireotide; PEG – pegvisomant.

Selected SOCs	Comparison	Evaluated ADRs for PAS	Other ADRs for targeted drug PAS	Evaluated ADRs for the drug used for comparison	Other ADRs for the drug used for comparison
Cardiac disorders	PAS-OCT	32	1615	927	31,375
	PAS-LAN	32	1615	205	7310
	PAS-PEG	32	1615	118	4724
	PAS-ALL	32	1615	1250	43,409
Endocrine disorders	PAS-OCT	53	1594	405	31,897
	PAS-LAN	53	1594	105	7410
	PAS-PEG	53	1594	84	4758
	PAS-ALL	53	1594	594	44,065
Gastrointestinal disorders	PAS-OCT	165	1482	3465	28,837
	PAS-LAN	165	1482	1001	6514
	PAS-PEG	165	1482	323	4519
	PAS-ALL	165	1482	4789	39,870
Hepatobiliary disorders	PAS-OCT	49	1598	1037	31,265
	PAS-LAN	49	1598	233	7282
	PAS-PEG	49	1598	135	4707
	PAS-ALL	49	1598	1405	43,254
Metabolism and nutrition disorders	PAS-OCT	289	1358	1490	30,812
	PAS-LAN	289	1358	340	7175
	PAS-PEG	289	1358	129	4713
	PAS-ALL	289	1358	1959	42,700
Vascular disorders	PAS-OCT	30	1617	1229	31,073
	PAS-LAN	30	1617	247	7268
	PAS-PEG	30	1617	118	4724
	PAS-ALL	30	1617	1594	43,065

Table S3 – Number of ADRs reported for gastro-intestinal ADRs. ALL – all other drugs excepted pasireotide; LAN – lanreotide, OCT – octreotide; PAS - pasireotide; PEG – pegvisomant.

Selected ADR	Comparison	Evaluated ADRs for PAS	Other ADRs for targeted drug PAS	Evaluated ADRs for the drug used for comparison	Other ADRs for the drug used for comparison
Abdominal distress	PAS-OCT	42	1605	1309	30,993
	PAS-LAN	42	1605	384	7131
	PAS-PEG	42	1605	77	4765
	PAS-ALL	42	1605	1770	42,889
Abnormalities in stool appearance and consistency	PAS-OCT	14	1633	198	32,104
	PAS-LAN	14	1633	91	7424
	PAS-PEG	14	1633	15	4827
	PAS-ALL	14	1633	304	44,355
Bowel movement disorders	PAS-OCT	66	1581	1905	30,397
	PAS-LAN	66	1581	559	6956
	PAS-PEG	66	1581	105	4737
	PAS-ALL	66	1581	2569	42,090
Vomiting	PAS-OCT	35	1612	552	31,750
	PAS-LAN	35	1612	163	7352
	PAS-PEG	35	1612	43	4799
	PAS-ALL	35	1612	758	43,901
Flatulence	PAS-OCT	8	1639	241	32,061
	PAS-LAN	8	1639	51	7464
	PAS-PEG	8	1639	5	4837
	PAS-ALL	8	1639	297	44,362
Pancreatitis (including acute and chronic)	PAS-OCT	11	1636	169	32,133
	PAS-LAN	11	1636	53	7462
	PAS-PEG	11	1636	12	4830
	PAS-ALL	11	1636	234	44,425

Table S4 – Number of ADRs reported for cholelithiasis. ALL – all other drugs excepted pasireotide; LAN – lanreotide, OCT – octreotide; PAS - pasireotide; PEG – pegvisomant,

Selected ADR	Comparison	Evaluated ADR for PAS	Other ADRs for targeted drug PAS	Evaluated ADR for the drug used for comparison	Other ADRs for the drug used for comparison
Stones affecting the hepatobiliary system	PAS-OCT	27	1620	344	30,649
	PAS-LAN	27	1620	91	7040
	PAS-PEG	27	1620	40	4725
	PAS-ALL	27	1620	475	42,414

Table S5 – Number of ADRs reported for hypotension. ALL – all other drugs excepted pasireotide; LAN – lanreotide, OCT – octreotide; PAS - pasireotide; PEG – pegvisomant.

Selected ADR	Comparison	Evaluated ADR for PAS	Other ADRs for targeted drug PAS	Evaluated ADR for the drug used for comparison	Other ADRs for the drug used for comparison
Hypotension	PAS-OCT	12	1635	190	30,803
	PAS-LAN	12	1635	40	7091
	PAS-PEG	12	1635	14	4751
	PAS-ALL	12	1635	244	42,645

Table S6 – Number of ADRs reported for adrenal insufficiency. ALL – all other drugs excepted pasireotide; LAN – lanreotide, OCT – octreotide; PAS - pasireotide; PEG – pegvisomant.

Selected ADR	Comparison	Evaluated ADR for PAS	Other ADRs for targeted drug PAS	Evaluated ADR for the drug used for comparison	Other ADRs for the drug used for comparison
Adrenal insufficiency	PAS-OCT	22	1625	15	30,978
	PAS-PEG	22	1625	7	4758
	PAS-ALL	22	1625	22	42,867

Table S7 – Number of ADRs reported for metabolic and nutritional disorders. ALL – all other drugs excepted pasireotide; LAN – lanreotide, OCT – octreotide; PAS - pasireotide; PEG – pegvisomant.

Selected ADRs	Comparison	Evaluated ADRs for PAS	Other ADRs for targeted drug PAS	Evaluated ADRs for the drug used for comparison	Other ADRs for the drug used for comparison
Electrolyte disorders	PAS-OCT	10	1637	116	30,877
	PAS-LAN	10	1637	21	7110
	PAS-PEG	10	1637	7	4758
	PAS-ALL	10	1637	144	42,745
Hypoglycemia	PAS-OCT	18	1629	262	30m731
	PAS-LAN	18	1629	65	7066
	PAS-PEG	18	1629	11	4754
	PAS-ALL	18	1629	338	42551
Glucose tolerance impaired	PAS-OCT	7	1640	19	30,974
	PAS-ALL	7	1640	26	42,863
Diabetes mellitus	PAS-OCT	103	1544	221	30,772
	PAS-LAN	103	1544	43	7088
	PAS-PEG	103	1544	44	4721
	PAS-ALL	103	1544	308	42,581
Type 2 diabetes	PAS-OCT	16	1631	39	30,954
	PAS-LAN	16	1631	14	7117
	PAS-PEG	16	1631	6	4759
	PAS-ALL	16	1631	59	42,830
Diabetic cetoacidosis	PAS-OCT	7	1640	6	30,987
	PAS-ALL	7	1640	8	42,881
